# A severe clinical phenotype of Noonan syndrome with neonatal hypertrophic cardiomyopathy in the second case worldwide with *RAF1* S259Y neomutation

**DOI:** 10.1017/S0016672319000041

**Published:** 2019-04-29

**Authors:** Hager Jaouadi, Amel Ben Chehida, Lilia Kraoua, Heather C. Etchevers, Laurent Argiro, Nadia Kasdallah, Sonia Blibech, Valérie Delague, Nicolas Lévy, Néji Tebib, Ridha Mrad, Sonia Abdelhak, Rym Benkhalifa, Stéphane Zaffran

**Affiliations:** 1 Biomedical Genomics and Oncogenetics Laboratory LR16IPT05, Institut Pasteur de Tunis, Université Tunis El Manar, Tunis, Tunisia; 2 Department of Pediatrics and Metabolic Diseases, La Rabta Hospital, Faculty of Medicine of Tunis, University of Tunis El Manar, Tunis, Tunisia; 3 Department of Congenital and Hereditary Diseases, Charles Nicolle Hospital, Faculty of Medicine of Tunis, University of Tunis El Manar, Tunis, Tunisia; 4 Aix Marseille Univ, INSERM, MMG, U1251, Marseille Medical Genetics, Marseille, France; 5 Neonatal Resuscitation and Intensive Care Unit of Military Hospital of Tunis, Military Hospital of Tunis, Tunisia; 6 Venoms and Therapeutic Biomolecules Laboratory LR16IPT08, Institut Pasteur de Tunis, Université Tunis El Manar, Tunisia

**Keywords:** hypertrophic cardiomyopathy, Noonan syndrome, *RAF1* mutation, RAS/MAPK pathway, whole exome sequencing

## Abstract

Noonan syndrome and related disorders are a group of clinically and genetically heterogeneous conditions caused by mutations in genes of the RAS/MAPK pathway. Noonan syndrome causes multiple congenital anomalies, which are frequently accompanied by hypertrophic cardiomyopathy (HCM). We report here a Tunisian patient with a severe phenotype of Noonan syndrome including neonatal HCM, facial dysmorphism, severe failure to thrive, cutaneous abnormalities, pectus excavatum and severe stunted growth, who died in her eighth month of life. Using whole exome sequencing, we identified a *de novo* mutation in exon 7 of the *RAF1* gene: c.776C > A (p.Ser259Tyr). This mutation affects a highly conserved serine residue, a main mediator of Raf-1 inhibition via phosphorylation. To our knowledge the c.776C > A mutation has been previously reported in only one case with prenatally diagnosed Noonan syndrome. Our study further supports the striking correlation of *RAF1* mutations with HCM and highlights the clinical severity of Noonan syndrome associated with a *RAF1* p.Ser259Tyr mutation.

## 1. Introduction

Noonan syndrome and related disorders (cardiofaciocutaneous syndrome [CFC], Costello syndrome [CS] and Noonan syndrome with multiple lentigines [formerly known as LEOPARD syndrome]) are autosomal dominant disorders characterized by a wide range of symptoms including facial dysmorphism, short stature, mental retardation and congenital heart defects often associated with hypertrophic cardiomyopathy (HCM) (Tartaglia *et al.*, 2011). These syndromes, along with Legius syndrome and type I neurofibromatosis, are collectively known as RASopathies and share overlapping phenotypic and molecular features, making accurate diagnosis challenging (Nystrom *et al.*, 2008; Tumurkhuu *et al.*, 2010).

The molecular basis of these disorders has been linked to mutations in components or regulators of the Ras/mitogen-activated protein kinase (MAPK) pathway, predominantly encoded by *PTPN11*, *BRAF*, *SOS1*, *HRAS*, *KRAS*, *MAP2K1*, *MAP2K2* and *RAF1* genes (Ko *et al.*, 2008; Ezquieta *et al.*, 2012; Search by Disease). The *RAF1* gene encodes a proto-oncogene serine/threonine-protein kinase of 648 amino acids. Structurally, the Raf-1 (also known as c-Raf) protein has three conserved regions (CR). Mutations identified in this gene are clustered in the CR2 domain, with only a few located in CR3 (Pandit *et al.*, 2007; Razzaque *et al.*, 2007). The CR2 domain is important for the regulatory phosphorylation and binding with the 14-3-3 consensus site.

Mutations located around Ser259 lead to decreased phosphorylation of the serine and dissociation from the 14-3-3 binding site, thus targeting the substrate to the catalytic domain in the CR3 domain (Kobayashi *et al.*, 2010). Functional analysis showed that dephosphorylation of Ser259 by *RAF1* mutations at this residue leads to extracellular signal-regulated kinases (ERK1 and ERK2) activation (Kobayashi *et al.*, 2010).

HCM is frequently observed in patients with RASopathies and might represent the major determinant in the outcome of these patients (Limongelli *et al.*, 2006). Interestingly, a striking correlation between *RAF1* mutations and HCM has already been described (Razzaque *et al.*, 2007; Ko *et al.*, 2008; Wilkinson *et al.*, 2012; Gelb *et al.*, 2015; Calcagni *et al.*, 2018). It has been noted that Noonan syndrome patients with HCM have a worse risk profile compared to patients with idiopathic or familial HCM (Prendiville *et al.*, 2014).

Here we report on a Tunisian patient with severe Noonan syndrome including neonatal HCM, leading ultimately to death. The aim of this study was to determine the genetic defect underlying the severe clinical phenotype of the patient.

## 2. Materials and methods

The parents provided their written informed consent to participate in this study. This work was conducted according to the principles of the Declaration of Helsinki and to the ethical guidelines of the institutions involved (Registration number: IRB00005445, FWA00010074). Genomic DNA was extracted from the samples according to standard techniques.

- Whole exome sequencing

Whole exome sequencing (WES) was performed for the parents, the affected child and her unaffected brother by the Genomics and Bioinformatics Platform (GBiM) of the INSERM U1251 Marseille Medical Genetics facility.

The samples were sequenced using library preparation protocols with the NimbleGen SeqCap EZ MedExome kit (Roche Sequencing Solutions, Madison, USA). The resulting libraries were subjected to paired-end sequencing on Illumina NextSeq 500 platform (Illumina, San Diego, CA, USA). Raw data were aligned against the human genome (hg19) using BWA 0.7.5. Variant calling and annotation were processed using GATK and ANNOVAR.

- Variant prioritization

Pedigree-based variant prioritization and co-segregation were performed with the Variant Annotation and Filtering Tool (VarAFT), version 2.12 (https://varaft.eu/). To pinpoint putatively pathogenic and causal variants we adopted the following filtering strategy: we first excluded variants with a minor allele frequency (MAF) >1% in the gnomAD database (http://gnomad.broadinstitute.org/). The remaining variants were filtered based on their type and genomic localization; thus, synonymous, intronic, variants in intergenic, 3′ and 5′ UTR regions were discarded. The obtained variants list was then filtered according to the *in silico* pathogenicity prediction. Thus, variants predicted as polymorphisms according to UMD-Predictor (http://umd-predictor.eu/), SIFT (http://sift.jcvi.org/), PolyPhen-2 (http://genetics.bwh.harvard.edu/pph2/), or Mutation Taster (http://www.mutationtaster.org/) were excluded. Subsequently, we searched for variants in the RAS/MAPK pathway by focusing on 20 genes previously associated with RASopathies (Table A1).

- Sanger validation

The selected variant was validated using PCR-based bidirectional Sanger sequencing.

## 3. Results

- Clinical presentation

The patient, a Tunisian girl, was the second child of apparently healthy and unrelated parents. She had a healthy 4-year-old brother. The mother and the father were respectively 33 and 39 years at conception. The family history was unremarkable. Pregnancy was uneventful. The child was delivered by caesarian section in the 40th week of pregnancy because of engagement failure. The Apgar scores were 9 at one minute and 10 at 5 minutes. Her birth measurements were normal (50–90th percentiles) (Table 1).

**Table 1.** Evolution of growth and echocardiographic features.

Age		Neonatal (first week)	5 months	8 months
Weight	Grams	3600	4300	3800
	Percentile	50–75th	<3rd	<3rd
Height	Centimeters	49.5	54	55
	Percentile	50th	<3rd	<3rd
Head circumference	Centimeters	37	40	41
	Percentile	90th	<3rd	<3rd
IVS		9 mm	10 mm	NA
PLVW		6 mm	8 mm	NA

IVS, interventricular septum thickness; NA, not available; PLVW, posterior left ventricular thickness.

**Fig. 1.**
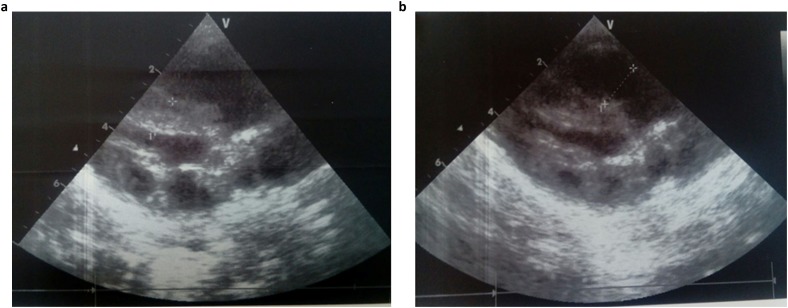
Echocardiogram at the age of 5 months showing concentric HCM (a) and right ventricular outflow tract dilation (b).

She was admitted 42 hours after birth in the neonatal department because of dyspnea, cardiac murmur and dysmorphic facial features. At 5 days, she was diagnosed with non-obstructive hypertrophic cardiomyopathy with a moderate pulmonary hypertension at 46 mmHg. She was treated with propranolol (4 mg/day). Transfontanellar and renal ultrasound examination were normal. Serum creatinine, thyroid function tests, ammonia and lactate levels were normal. Complete blood count revealed hypochromic anemia treated with iron therapy. The mother's HbA1c was normal (5.1%). R-banded chromosome analysis on cultured peripheral blood lymphocytes from the patient was normal 46,XX. The chest computed tomography scan, carried out at 12 days, ruled out coarctation of the aorta. Rhythmic Holter, carried out at 40 days, showed a normal sinus rhythm with heart rate of 133 beats per minute and increased P wave amplitude. Echocardiography controlled at 5 months showed concentric asymmetric hypertrophy of the ventricles and interventricular septum leading to a mild right ventricular outflow tract obstruction (maximum gradient between pulmonary artery and right ventricle = 16 mm) (Figure 1 & Table 1). A patent foramen ovale and a moderate dynamic mitral insufficiency were also noted.

**Fig. 2.**
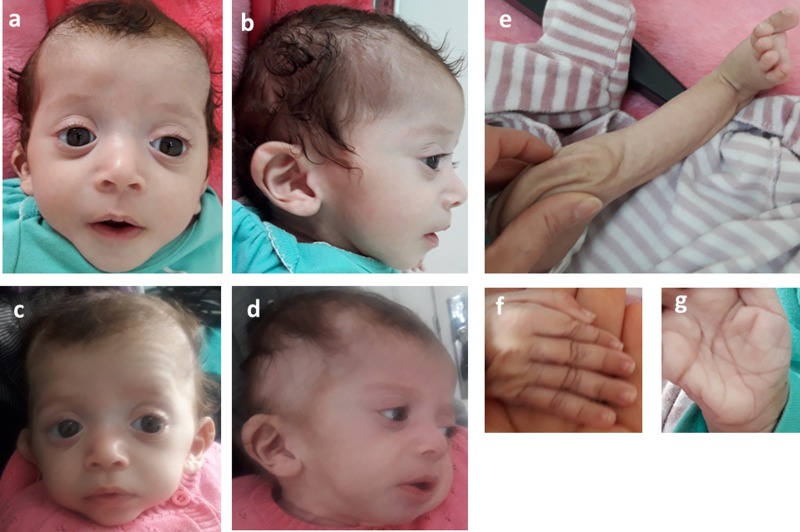
Photographs of the patient at 5 months (a, b, e, f, g) and 6 months (c, d): note the dysmorphic facial features (a, b, c, d) including large forehead, frontal bossing, bitemporal narrowing, shallow orbital ridge, hypertelorism, exophthalmos, down-slanting palpebral fissures, depressed root of nose and bulbous tip, anteverted nares, low-set, posteriorly rotated ears with thickened helix (b, d), smooth long philtrum (a) becoming deeply grooved (c), small mouth, thickening of lips (c), full cheeks (c) and retrognathia (b, d) and the cutaneous abnormalities including sparse hair, eyebrows and eyelashes (a, b, c, d), redundant and loose skin on body members (e), hands and feet (f), and deep palmoplantar creases (g).

Consequently, the girl was referred to a pediatric and metabolic department for further investigations. Clinical evaluation by pediatricians and geneticists found a severe failure to thrive (Table 1). The dysmorphic facial features including large forehead, frontal bossing, bitemporal narrowing, shallow orbital ridge, hypertelorism, exophthalmos, down-slanting palpebral fissures, depressed root of nose and moderate bulbous tip, anteverted nares, low-set, posteriorly rotated ears with thickened helix, smooth long philtrum, small mouth, thin lips, retrognathia and a short neck with excess nuchal skin (Figure 2). Cutaneous abnormalities were remarkable including sparse hair, eyebrows and eyelashes, redundant and loose skin on body members, hands and feet, and deep palmoplantar creases (Figure 2). A pectus excavatum and umbilical hernia were also noted. Heart auscultation indicated systolic murmur without features of heart failure. Neurologic examination showed axial and peripheral hypertonia with large joint stiffness. Metabolic investigations (lactate cycle, plasma free and total carnitine levels, chromatographic analysis of amino acids and organic acids) were normal. The patient was diagnosed with Noonan syndrome or CFC, as key features of these syndromes were present, namely the characteristic facies, the failure to thrive, the HCM, the pectus excavatum and the cutaneous abnormalities. Molecular testing for a germline RASopathy was indicated.

At the age of 6 months, at blood sampling, the patient showed some changes in facial appearance; the philtrum became deeply grooved, the lips thicker, the nose bulbous and the cheeks full, which were most suggestive of Noonan syndrome (Figure 2).

On the last evaluation, at 8 months, she had a severe stunted growth (Table 1), more evident dysmorphic face, cardiac murmur, mild hepatomegaly, normal psychomotor development and a normal pulmonary examination. However, she died at home of a respiratory infection a few days later.

- Whole exome sequencing

Considering the overlapping features between Noonan and CFC syndrome in our patient, her severe clinical profile and the genetic heterogeneity of RASopathies, we performed exome sequencing of the patient, her unaffected brother and both parents to identify the disease-causing mutation. WES data from the family were simultaneously analysed and segregated using VarAFT software (Desvignes *et al.*, 2018). The filtering strategy is detailed in the ‘Materials and Methods’ section.

By focusing on 20 genes of the RAS/MAPK pathway, only one exonic variant in exon 7 of the *RAF1* gene was found. All remaining variants were intronic. All variants in the RAS/MAPK genes identified in the affected child are listed in the Supplementary Material (second sheet). The *RAF1*: c.776C > A; p.Ser259Tyr missense variant occurred *de novo*. The genomic coordinates of this variant in the human assembly GRCh37/hg19 is chr3:12,645,693-12,645,693.

To review the mutation spectrum of Noonan syndrome and specifically the *RAF1* gene, the European Network on Noonan Syndrome and related disorders was queried. The *PTPN11* gene is the most implicated gene in Noonan syndrome (61%), followed by *SOS1* and *RAF1* genes (15 and 6%, respectively). Approximately 91% of *RAF1* variants are associated with Noonan syndrome (Search by Disease). Moreover, six variant alleles were reported at the Ser259 residue (Table 2). The p.Ser259Tyr has only been reported once in a fatal case of Noonan syndrome (Hakami *et al.*, 2016).

**Table 2.** Allelic heterogeneity of the p.Ser259 residue of *RAF1* gene.

c.DNA	Protein	Classification	Count	Phenotype	Reference
c.776C > G	p.Ser259Cys	Mutation	2	Noonan syndrome	Unpublished
c.776_777delinsTA	p.Ser259Leu	Mutation	1	LEOPARD	Kuburović *et al.*, 2011
c.775T > A	p.Ser259Thr	Mutation	1	Noonan syndrome	Lee *et al.*, 2011
c.776C > T	p.Ser259Phe	Mutation	2	LEPOARD/Noonan syndrome	Pandit *et al.*, 2007
c.775T > C	p.Ser259Pro	Mutation	2	Noonan syndrome	Unpublished
c.776C > A	p.Ser259Tyr	Mutation	1	Noonan syndrome	Hakami *et al.*, 2016

To assess the functional impact of the p.Ser259Tyr variant, several *in silico* prediction tools were used (Table 3).

**Table 3.** Indications of pathogenicity of the p.Ser259Tyr mutation.

*In silico* prediction tool	Prediction
CADD-score (Combined Annotation Dependent Depletion)	26.1
LRT (Likelihood Ratio Test)	Deleterious
Mutation Taster	Disease causing
UMD-Predictor	Pathogenic
PolyPhen-2 (Polymorphism Phenotyping v2)	Damaging
Provean (Protein Variation Effect Analyzer)	Damaging
SIFT (Sorting Intolerant From Tolerant)	Damaging

Moreover, the *RAF1* c.776C > A; p.Ser259Tyr is absent from the following databases: gnomAD (http://gnomad.broadinstitute.org), 1000 Genomes (http://www.internationalgenome.org), and GME Variome (http://igm.ucsd.edu/gme/). Therefore, no frequency data were available. The variant was also absent from our in-house database, gathering WES data from 70 Tunisian individuals (140 chromosomes).

**Fig. 3.**
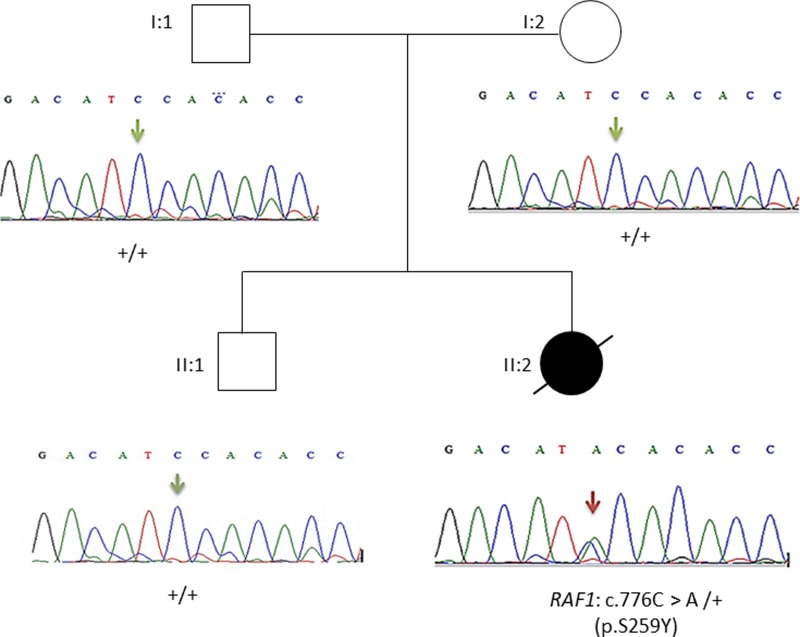
Pedigree of the family. Affected proband is denoted by filled circle, unaffected members are denoted by empty symbols. Sequence electropherograms are shown below symbols. (+) indicates the wild-type allele, the arrow indicates the position of the mutation.

This result as well as the mode of transmission were validated by Sanger sequencing. The electropherograms shown in Figure 3 confirmed the p.Ser259Tyr mutation and the mode of inheritance.

Overall, the heterozygous *RAF1* p.Ser259Tyr variant identified by WES confirms the diagnosis of Noonan syndrome in our patient.

## 4. Discussion

We report on an 8-month-old girl affected with Noonan syndrome and neonatal HCM. She had a severe clinical phenotype, resulting in a fatal outcome. The clinical presentation included neonatal HCM, facial dysmorphism, severe failure to thrive, cutaneous abnormalities and pectus excavatum. These clinical and dysmorphic facial features were suggestive of Noonan syndrome. Nevertheless, the patient had remarkable dermatological features including sparse hair, eyebrows and eyelashes, redundant skin and deep palmoplantar creases. Of note, sparse hair, sparse eyelashes and cutaneous abnormalities can be pronounced in CFC (Lee *et al.*, 2011).

WES was performed and showed a *de novo* p.Ser259Tyr mutation in exon 7 of *RAF1*. RAF1 or CRAF is an entry point to the RAS/MAPK pathway. It acts as a downstream effector of RAS signalling alongside BRAF (Dhillon *et al.*, 2002). *RAF1* activation initiates a MAPK cascade that comprises a sequential phosphorylation of the dual-specific MAPK kinases MEK1/MEK2 (encoded by the *MAP2K1* and *MAP2K2* genes) followed by the extracellular signal-regulated kinases ERK1 and ERK2 (encoded by *MAPK3* and *MAPK1*, respectively) (Dhillon *et al.*, 2002; Pandit *et al.*, 2007). The conserved region CR2 of RAF1 plays a key role in its activation. Interestingly, the majority of *RAF1* mutations are located in the CR2 domain. Functional characterization showed that the *in vitro* activity of RAF1 proteins with mutations in the CR2 domain are higher than the activity of normal RAF1 in the presence of growth factor (Lee *et al.*, 2011). Activation and inactivation states of RAF1 are regulated by the phosphorylation of several serine and threonine residues. In its inactive conformation, the N-terminal region of RAF1 interacts with the kinase domain at the C-terminal region and leads to its inactivation. This conformation is stabilized by the consensus 14-3-3 recognition sequence that binds to phosphorylated Ser259 and Ser621 (Pandit *et al.*, 2007). It has also been demonstrated that *RAF1* mutants in the CR2 domain impaired phosphorylation of Ser259, abrogated the binding to the 14-3-3 site and lead to a partial activation of ERK (Kobayashi *et al.*, 2010; Tumurkhuu *et al.*, 2010). Thus, the lack of phosphorylation of Ser259 is the primary pathogenic mechanism in activating *RAF1* mutants (Dhillon *et al.*, 2002; Kobayashi *et al.*, 2010). Gain-of-function mutations in the *RAF1* gene lead to constitutive activation of the RAS/MAPK pathway (Hopper *et al.*, 2015).

Gain-of-function mutations in *RAF1* were identified in 3–17% of patients with Noonan syndrome and two patients with Noonan syndrome with multiple lentigines (Pandit *et al.*, 2007; Razzaque *et al.*, 2007). In a case series study reporting 212 newborns with clinical suspicion of Noonan syndrome and related disorders, the *RAF1*; p.Ser259Tyr mutation was reported in one patient with Noonan syndrome (Hakami *et al.*, 2016). No clinical data of this patient were provided, except a severe edema detected by ultrasonography (Hakami *et al.*, 2016). Therefore, our patient is the second case reported in the literature carrying the p.Ser259Tyr mutation. However, allelic heterogeneity at Ser259 residue was noted (Table 2). As an illustration, the p.Ser259Thr mutation was functionally characterized by assaying the activation status of the downstream effectors, MEK2 and ERK2. In the presence of epidermal growth factor stimulus, a higher level of phosphorylated MEK1 and ERK2 was observed in cells expressing p.Ser259Thr than in those expressing wild-type *RAF1* (Lee *et al.*, 2011).

Our patient had neonatal non-obstructive HCM with mild pulmonary hypertension. Previous studies noted that HCM in Noonan syndrome arises early in life, with a median age of 5 months (Hickey *et al.*, 2011; Wilkinson *et al.*, 2012). HCM might represent the major determinant in the outcome of these patients (Limongelli *et al.*, 2006; Wilkinson *et al.*, 2012), particularly in patients with early onset of HCM (Calcagni *et al.*, 2018). HCM and pulmonic stenosis are the most common cardiac abnormalities in *RAF1* mutation carriers (Kobayashi *et al.*, 2010). Sudden deaths in patients with *RAF1* mutations has been likely associated with heart abnormalities and their complications (Kobayashi *et al.*, 2010; Wilkinson *et al.*, 2012). The molecular pathogenesis of HCM in RASopathies results from hyperactivation of several signalling pathways (Calcagni *et al.*, 2018). Pandit *et al.* noted that Noonan syndrome patients with HCM carried gain-of-function *RAF1* mutations resulting in increased ERK activation, whereas Noonan syndrome patients without HCM harbour loss-of-function *RAF1* mutations (Pandit *et al.*, 2007). These findings suggest that enhanced ERK activation may underlie cardiomyocyte hypertrophy.

Interaction of signal transduction pathways such as the MAPK pathway and their activators may underlie cardiac hypertrophy (Rohini *et al.*, 2010). The exposure of cardiomyocytes to stress leads to the activation of small G proteins such as Ras and Raf, which further activates MAPK signalling. Hence, the activation of the RAS/RAF/MEK/MAPK cascade is an integral part of the pathogenesis of HCM (Sala *et al.*, 2012). Of note, *in vivo* inhibition of MEK attenuates cardiac growth in both induced and genetic models of hypertrophy (Armstrong, 2004; Sala *et al.*, 2012).

At 5 months, echocardiography of our patient revealed a concentric asymmetric hypertrophy of the ventricles and interventricular septum. Indeed, biventricular hypertrophy has been noted in patients with Noonan syndrome carrying *RAF1* mutations (Sana *et al.*, 2014; Thompson *et al.*, 2017). Moreover, HCM in RASopathies is characterized by asymmetrical hypertrophy with major involvement of basal interventricular septum (Calcagni *et al.*, 2018). Altogether, the clinical presentation described in this study and the *in silico* prediction of the functional impact of *RAF1* p.Ser259Tyr mutation strengthens its claim to pathogenicity.

In conclusion, WES allowed us to identify a *de novo* p.Ser259Tyr mutation in *RAF1* and to provide a definite diagnosis of Noonan syndrome. Differential diagnosis of Noonan syndrome and related disorders is relevant due to their different management and prognosis as well as the resulting genetic counselling. In the present family, as the p.Ser259Tyr mutation occurred *de novo*, the risk for future pregnancies is low (<1%). Moreover, no medical follow-up will be required for their healthy second son. This report further supports the implication of *RAF1* mutations in HCM pathogenesis and highlights the correlation of p.Ser259Tyr mutation with a severe phenotype in Noonan syndrome. WES can improve specialized counselling, allowing focused and brief forms of psychological assistance in a timely manner which can play a central role in the management of psychological distress and in ensuring the relative well-being of the family.

## Supplementary material

For supplementary material accompanying this paper visit https://doi.org/10.1017/S0016672319000041. click here to view supplementary material
